# 

**Published:** 1891-03-21

**Authors:** 


[illegible]o. 234, Vol. IX.—March 21st, 1891.

[R]egistered at the General Post Office as a Newspaper for transmission in the United Kingdom and Abroad.]

WITH EXTRA NURSING SUPPLEMENT.
Per Annum, 6s. 6d.; post free.
Monthly Parts, 6d.; post free 8d.
Ditto per Annum, 8s., post free.

### THE HOSPITAL

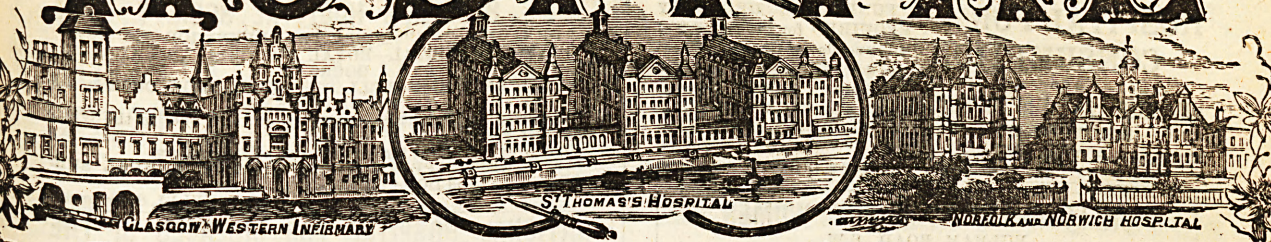

A WEEKLY JOURNAL OF

### Science, Medicine, Nursing and Philanthropy.

### SATURDAY, MARCH 21st, 1891.

**The College of Physicians in a New Character** - 355
**Passing Topics.**—An Unhappy Juxtaposition - 356
**The Doctor's Armchair.**—A Feat to be Considered - 357
**The Microscope in Medicine.** By FRANK J. WETHERED, M.D.—X.—The Examination of Urinary Deposits - 358
**Everybody's Page** - 359
**Notes and News** - 360
**Scraps and Gleanings** - 361
**Annotations.**—The Suppression of Juvenile Smoking—Charity and Charity Organisation—Hygiene and Demography - 362
**Books Received** - 365
**The Practitioner's Mirror and Retrospect:—**
MEDICINE.—On the Therapeutical Action of Koch's Remedy 363
SURGERY.—Torticollis - 364
THERAPEUTICS.—Rhus Toxicodendron - 364
NEW DRUGS, APPLIANCES, AND THINGS MEDICAL.—Dr. Strochem's Hypodermic Syringe—Kond's Euonymised Cocoa—Peterman's Cockroach and Beetle Food (non-poisonous)—Dr. Angell's Food for Infants and Invalids - 365
**Metropolitan Hospital for the Insane.** By FRANCIS H. WALMSLEY, M.D.—**The Lords' Committee** - 366
**The Student of Medicine.**—Notes from the Schools—Appointments—Vacancies—Pass Lists—Athletics.

### EXTRA SUPPLEMENT—THE NURSING MIRROR.

**En Passant.**—Advance America—A Nurse Aged Sixteen—Short Items—The Spirit of Charity—Haggerston and Hoxton—An Ancient Remedy—East London Nursing Association - cxxxvii

**Lectures on Surgical Ward Work and Nursing.** By ALEXANDER MILES, M.B.Edin., C.M., F.R.C.S.E. Lecture XVII.—Preparation of Patient for Operation - cxxxviii

**The Eastern Hospital Scandal** - cxxxix

**For Reading to the Sick.**—Help in Suffering - cxxxix

**The Royal National Pension Fund for Nurses** - cxl

**Princess Christian's Daughter** - cxl

**Jottings from "Gib."** - cxli

**Everybody's Opinion.**—Nurses' Home, Truro—A Nurse's Hours - cxli

**Presentations—Notes and Queries** - cxli

**Off Duty.**—Jarvey's Band - cxlii

**Appointments** - cxlii

**Amusements and Relaxation** - cxlii

